# Supplementary material for: Influence of Immunosuppressive Regimen on Diffusivity and Oxygenation of Kidney Transplants—Analysis of Functional MRI Data from the Randomized ZEUS Trial
Source: J Clin Med. 2022 Jun 8;11(12):3284. doi: 10.3390/jcm11123284 (PMC9224619; doi:10.3390/jcm11123284)
Supplement: Supplementary file 1 [file jcm-11-03284-s001.zip › Table S1.pdf]

Table S1. Clinical characteristics of the total study population (*n*=16).

	**Subjects included in final MR analysis** (*n*=12)	**Subjects excluded from final MR analysis** (*n*=4)
Age at transplantation (years)	45±11 (47; 29-62)	54.5±3 (54; 52-59)
Female gender (%)	50	25
Living donor (%)	17	0
Donor age (years)	51±14 (56; 23-65)	52±10 (52; 42-63)
Extended criteria donor (%)	33	25
Transplant episode (%)		
- first	69	75
Cold ischemia time (min.)	467±232	449±190
Delayed graft function (%)	8	25
eGFR (ml/min/1.73 m^2)	64±22 (59; 35-109)	63±4 (65; 58-66)
CsA trough level (ng/ml)		
- at baseline	167±57 (144; 87-241)	154±8 (157; 145-161)
- at 12 months	131±6 (131; 126-135)	140±24 (132; 120-167)
24 hour-blood pressure (mmHg)		
- systolic	119±12	129±4
- diastolic	78±11	83±5
- mean	99±12	106±5
Hemoglobin (g/l)		
- at baseline	124±16	149±30
- at 12 months	121±16	151±28
Biopsy findings at screening		
- BPAR (%)	0	25
- arteriolar hyalinosis (0-3)	0.4±0.8	0.7±1.2
- IFTA (0-3)	0.5±0.5	0.7±0.6
Time to MR scan (months)		
- baseline MR	3.8±0.6	3.5±0.6
- second MR	12±0.7	na

Values are given as mean ± standard deviation (median; range) or as percentage of patients, as appropriate. Number (n); ciclosporin (CsA); everolimus (EVE); estimated glomerular filtration rate (eGFR) estimated according to chronic kidney disease epidemiology collaboration formula; magnetic resonance (MR); biopsy-proven acute rejection (BPAR); interstitial fibrosis and tubular atrophy (IFTA); not available (na).
