# Supplementary material for: Influence of Immunosuppressive Regimen on Diffusivity and Oxygenation of Kidney Transplants—Analysis of Functional MRI Data from the Randomized ZEUS Trial
Source: J Clin Med. 2022 Jun 8;11(12):3284. doi: 10.3390/jcm11123284 (PMC9224619; doi:10.3390/jcm11123284)
Supplement: Supplementary file 1 [file jcm-11-03284-s001.zip › Table S2.pdf]

Table S2. Histological characteristics at baseline and follow-up.

Subjects	Randomization	AR	Arteriolar hyalinosis	ATN	IFTA	Other	Precision other findings
Patient 1*	CsA	0/0	2/1	0/0	1/1	1/0	Interstitial-intratubular calcium crystals
Patient 2*	CsA	na	na	na	na	na	
Patient 3	CsA	0/1	1.5/2	0/0	1/1	0/1	Borderline rejection, BKV
Patient 4	CsA	0/1	0/0	0/0	1/0	1/0	Severe arteriosclerosis, cytoplasmic crystals/0
Patient 5	CsA	0/0	0/0	0/1	0/0	0/1	Interstitial calcium crystals
Patient 6*	CsA	0/na	0/na	0/na	0/na	0/na	Glomerulomegaly
Patient 7	CsA	0/0	0/2	0/0	0/1	0/0	
Patient 8	EVE	0/0	0/0	0/0	1/1	0/0	
Patient 9	EVE	na/na	na/na	na/na	na/na	na/1	Soft tissue (no renal tissue)
Patient 10	EVE	na/na	na/na	na/na	na/na	na/na	Muscle (no renal tissue)
Patient 11	EVE	na/0	na/0	na/0	na/1	1/0	Muscle (no renal tissue)
Patient 12	EVE	0/0	2/3	0/0	1/0	1/0	Calcium crystals
Patient 13	EVE	na/na	na/na	na/na	na/na	na/na	
Patient 14	EVE	0/1	0/0	0/0	0/0	0/1	n.r.; interstitial concrements, severe arteriosclerosis, borderline rejection
Patient 15	EVE	0/0	0/0	0/0	0/1	0/0	
Patient 16*	na	1/na	0/na	0/na	1/na	0/na	

Values are given as raw data for two different time-points (month 4.5/month12) on a semiquantiative scale (0-3 with increasing severity) for arteriolar hyalinosis and interstitial fibrosis and tubular atrophy (IFTA) or binary (0=absence, 1=presence) for acute rejection (AR), acute tubular necrosis (ATN) and other findings. Time-points are separated ciclosporin (CsA); everolimus (EVE); not available (na); not representative (n.r.).* designs patients excluded from final MR analysis.
